# Picosecond pump–probe X-ray scattering at the Elettra SAXS beamline

**DOI:** 10.1107/S1600577519015728

**Published:** 2020-01-01

**Authors:** Max Burian, Benedetta Marmiroli, Andrea Radeticchio, Christian Morello, Denys Naumenko, Giorgio Biasiol, Heinz Amenitsch

**Affiliations:** a Institute of Inorganic Chemistry, Graz University of Technology, Stremayrgasse 9/V, 8010 Graz, Austria; b Elettra-Sincrotrone Trieste SCpA, Strada Statale 14, km 163.5, Basovizza, TS 34149, Italy; c Laboratorio TASC, CNR-IOM at Area Science Park, Strada Statale 14, km 163.5, Basovizza, TS 34149, Italy

**Keywords:** pump–probe experiments, transient X-ray scattering

## Abstract

A new setup for picosecond pump–probe X-ray scattering at the Austrian SAXS beamline at Elettra-Sincrotrone Trieste is presented. A high-power/high-repetion-rate laser has been installed on-site, delivering UV/VIS/IR femto­second-pulses in-sync with the storage ring. Data acquisition is achieved by gating a multi-panel detector, capable of discriminating the single X-ray pulse in the dark-gap of the Elettra hybrid filling mode. Specific aspects of laser- and detection-synchronization, on-line beam steering as well protocols for spatial and temporal overlap of laser and X-ray beam are also described. The capabilities of the setup are demonstrated by studying transient heat-transfer in an In/Al/GaAs superlattice structure and results are confirmed by theoretical calculations.

## Abstract 2

A new setup for picosecond pump–probe X-ray scattering at the Austrian SAXS beamline at Elettra-Sincrotrone Trieste is presented. Details of the specific implementation are discussed and results of a first transient-heating experiment are shown.

## 1. Introduction   

One of the unique properties of synchrotron light, besides its high brilliance and tunable wavelength, is its pulsed nature: electrons in buckets revolve in the storage ring and emit radiation in picosecond pulses (Silly *et al.*, 2017 ▸; Schotte *et al.*, 2001 ▸; Winick & Doniach, 1980 ▸). Common time-resolved experiments do not observe and benefit from this temporal structure of light, as acquisition times are usually in the microsecond to millisecond regime, *i.e.* several orders of magnitude larger than a single radiation pulse (Ghazal *et al.*, 2016 ▸; Marmiroli *et al.*, 2009 ▸; Oka *et al.*, 2005 ▸). Stroboscopic experiments, on the other hand, thrive from the outstanding time resolution given by the radiated pulse length: a physical/chemical process is initiated synchronous with the storage ring revolution frequency such that each radiation pulse takes a snapshot of the ongoing reaction (Wulff *et al.*, 1997 ▸; Zinin, 1983 ▸; Reusch *et al.*, 2014 ▸; Zolotoyabko *et al.*, 2003 ▸). These single-pulse experiments may hence be seen as the next generation of time-resolved experiments – a driving force that motivates the upgrade of many operational synchrotron facilities towards more brilliant and more compact pulses (Horejs, 2018 ▸).

In the past decade, commercial femtosecond laser systems have become stable, powerful and cost effective sources of UV/VIS/IR light (Sibbett *et al.*, 2012 ▸; Keller, 2010 ▸). When synchronized to the bucket structure of a storage ring (Jo *et al.*, 2014 ▸), these laser pulses pump ultrafast phenomena in condensed matter, whereas the time-delayed synchrotron radiation probes the transient sample response (Schiwietz *et al.*, 2016 ▸; Haldrup *et al.*, 2012 ▸; Wuilleumier & Meyer, 2006 ▸; Fournier & Coppens, 2012 ▸). Which physical or chemical phenomenon is tracked during the experiment depends on the wavelength of the probe pulse. Here, scattering of picosecond pulses in the hard or medium X-ray regime offers the unique possibility to capture light-induced structural motion at the atomic level, such as, for example, crystalline lattice vibrations (Briggs *et al.*, 2019 ▸; Gaal *et al.*, 2014 ▸), nano-scale heat transport (Plech *et al.*, 2004 ▸; Shayduk *et al.*, 2016 ▸) and molecular re­arrangement (Ihee *et al.*, 2005 ▸; Cammarata *et al.*, 2008 ▸). While alternative sources such as free-electron lasers (McNeil & Thompson, 2010 ▸; Helml *et al.*, 2014 ▸) or laboratory setups (Weisshaupt *et al.*, 2014 ▸; Bargheer *et al.*, 2004 ▸) of picosecond (or shorter) X-ray pulses are generally available, they are either difficult to access or provide only low flux compared with synchrotron radiation. Consequently, an increasing number of medium/hard X-ray scattering beamlines around the world have expanded their portfolio towards laser-pumped experiments, addressing new scientific questions (March *et al.*, 2011 ▸; Wang *et al.*, 2017 ▸; Navirian *et al.*, 2012 ▸; Enquist *et al.*, 2018 ▸; Wulff *et al.*, 2003 ▸; Sun *et al.*, 2016 ▸).

In this work, we present a new setup for picosecond laser-pump/X-ray-probe experiments at the Austrian small-angle X-ray scattering (SAXS) beamline of the Elettra synchrotron. Similar to other available setups, we pursue the strategy of transient measurements at high repetition rates, which drastically increases the effective X-ray flux at the sample. Such repetition rates in the >100 kHz regime require repeatable discrimination of single-bunch radiation, which we achieve by electronic gating of a multi-panel X-ray detector and hence avoid installation of additional elements in the beamline front-end such as a chopper. We further developed an ‘on-line table’: a dedicated module with all optical elements for laser-pumped experiments that may simply be placed on the beamline frame and allows rapid transition between experimental conditions. The capabilities of the new setup are shown in a reference experiment that studies transient heating in a custom In/Al/GaAs superlattice.

## 2. Experimental setup   

### 2.1. Elettra storage ring – the hybrid filling mode   

**Figure 1:**
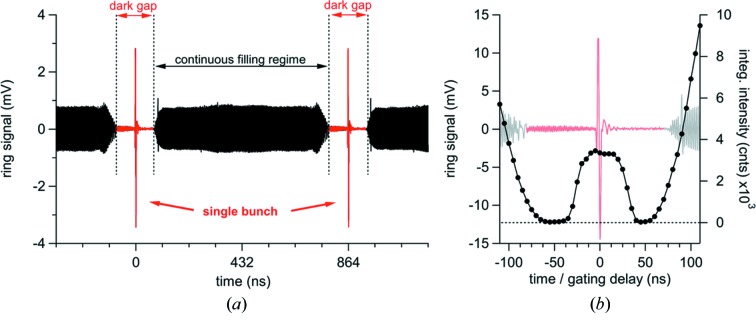
(*a*) Ring signal of the Elettra storage ring in hybrid filling mode, consisting of (i) a 704 ns continuous filling regime (black), (ii) a 160 ns dark gap (red) and (iii) a single electron bunch in the dark gap centre (see arrow). (*b*) Magnification of the ring signal close to the dark gap region (grey, light red) compared with the integrated intensity of the Pilatus3 1M (Dectris, Switzerland) X-ray detector (black dots) when scanning the gating delay.

The third-generation Elettra storage ring operates at either 2 GeV (310 mA) or 2.4 GeV (160 mA) energy (current) with four cavities running at approximately 500 MHz (2 ns) – the radio frequency (RF) bucket clock. Along the ring circumference, electron bunches may be placed within 432 buckets spaced by 2 ns, resulting in a circumference time of 864 ns – the 1.157 MHz ring clock (RC). By selectively filling these 432 buckets with electron bunches, the temporal emission function at any given insertion device may be controlled. The most attractive of these bunch structures is the ‘hybrid filling mode’, consisting of [see Fig. 1 ▸(*a*) ▸]: (i) a 704 ns ‘continuous filling’ regime [352 bunches with approximately 0.9/0.5 mA (2/2.4 GeV)], (ii) a 160 ns ‘dark gap’ or camshaft (80 buckets) and (iii) a single bunch in the camshaft centre [with approximately 3.5 mA (2/2.4 GeV)] (Karantzoulis *et al.*, 2018 ▸). This hybrid filling mode has no mentionable drawback in radiation flux for other beamlines and at the same time allows for transient ‘pulsed’ experiments by isolation of the camshaft single-bunch. The time-domain resolution of such transient measurements is limited by the temporal width of the single bunch, which is approximately 250 ps full width at half-maximum (FWHM) at 3.5 mA (Stebel *et al.*, 2011 ▸; Moise *et al.*, 2008 ▸; Karantzoulis *et al.*, 2014 ▸).

### 2.2. Beamline and X-ray detection   

The SAXS beamline at the Elettra storage ring has been operational for more than 20 years (Amenitsch *et al.*, 1995 ▸, 1998 ▸) and has been optimized for its flexible sample environment. Over these past two decades, a wide range of experiments have been successfully carried out in both transmission and grazing-incidence geometry, including, for example, microfluidic (Marmiroli *et al.*, 2010 ▸), electrochemical (Prehal *et al.*, 2017 ▸), thermal (Rath *et al.*, 2019 ▸) and even IR laser-flash (Yaghmur *et al.*, 2010 ▸) measurements. As, however, experiments utilizing the pulsed nature of the Elettra storage ring are unprecedented at the SAXS beamline, considerations regarding the beamline layout and infrastructure as well as its adequacy for transient experiments have to be made.

A detailed description of the beamline may be found in the literature (Amenitsch *et al.*, 1998 ▸) – only a brief summary is given here. The SAXS beamline is situated downstream of the shared W14.0 insertion device at exit 5.2: a 4.5 m multipole wiggler with an effective source size of 3.9 mm × 0.3 mm (H × V). A radiation cone with an acceptance of 1.5 mrad × 0.3 mrad (H × V) is then monochromated to either 5.4, 8 or 16 keV and is further focused to a maximum spot size of 1.2 mm × 0.6 mm (H × V, FWHM). Using the flexible arrangement of up to three pairs of collimation slits (spaced approximately 1.5 m) as well as additional pinholes, spot sizes down to 20 µm may be achieved. For laser-pumped experiments, X-ray spot sizes of approximately 200 µm × 200 µm are most feasible. At this size, the total flux at the sample is approximately 10^10^ photons s^−1^, which corresponds to a maximum single-bunch flux at the sample of approximately 10^8^ photons s^−1^ (considering radiation of single bunches only at the maximum possible repetition rate of 1.157 MHz). Most experiments are, however, not carried out at full repetition rate but only at a fraction of the ring clock, as most samples need to thermalize and/or return to ground state between optical pumps to avoid immediate laser damage. Using, for example, the single pulse from every fourth repetition would hence mean that 0.25% of the X-rays on the sample are actually counted on the detector – this would be a meaningful timing configuration for solid-state samples. For liquid jet experiments (which we foresee in the future), repetition rates above 100 kHz (approximately every 12th ring repetition) are not feasible due to the massive required sample volumes. Below 100 kHz, less than 0.08% of the X-rays on sample would be counted on the detector, such that these experiments would hence strongly benefit from a major storage ring and beamline improvement, as foreseen in the upcoming Elettra upgrade.

**Figure 2:**
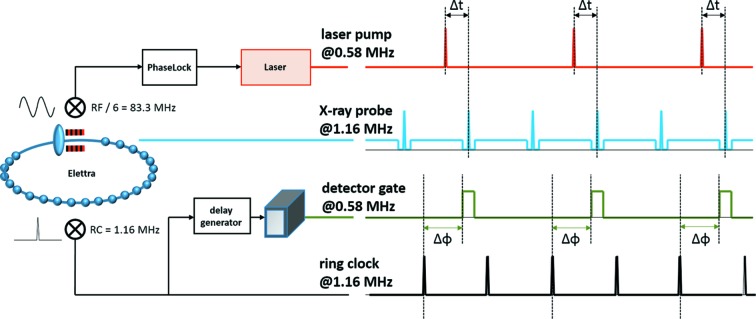
Synchronization scheme of electronic and optical components to the storage ring time-base: the radio frequency (RF) and ring clock (RC) signals. Here, ΔΦ describes the gating delay to isolate the X-ray scattering from the single bunch and Δ*t* describes the delay between laser-pump and X-ray probe. See text for further details.

X-ray detection is achieved using a Pilatus3 1M (Dectris, Switzerland) area detector, which may be operated in a ‘gated-configuration’ (Ejdrup *et al.*, 2009 ▸; Kraft *et al.*, 2009 ▸): a 20 ns TTL pulse activates the detector for approximately 120 ns, which allows for discrimination of scattering contributions of the ‘continuous filling regime’, thereby selecting the scattering of the single-pulse only. A digital delay generator (P400, Highland-Electronics, USA) which is triggered by the ring clock (1.157 MHz) generates the gating signal with tunable length and delay (see Fig. 2 ▸, black and green trace). The gating delay depends on specific attributes of the involved electronic devices and components and therefore it has to be determined prior to the experiment by a simple delay scan (see Fig. 2 ▸, ΔΦ). The ideal gating delay is found by tracking the integrated detector intensity, for example, of air-scattering, as shown in Fig. 1(*b*) ▸ (the time-overhead for such a scan is approximately 2 min).

While single-bunch X-ray detection by electronic gating is a resource-efficient approach, one has to consider possible limitations in detector response and/or counting efficiency resulting from the high X-ray fluence in the single bunch. Generally, when a single photon is counted on the detector pixel, the affected pixel is paralysed (non-counting) until the generated charge is collected. Multiple X-rays on a single pixel can hence only be counted when coming from different bunches, introducing a ‘single X-ray per pixel and bunch’ limit. The ‘instant re-trigger technology’ implemented in the Pilatus3 (Dectris, Switzerland) detectors used in this work reduces this problem, especially for single-bunch filling modes (Trueb *et al.*, 2015 ▸). However, it should be noted that the intensity on the detector is only in very rare cases of the order of the counting limit. Considering the realistic flux for pump–probe experiments at the Austrian SAXS beamline of 10^10^ photons s^−1^, this would mean that each single pulse contains approximately 80 photons, such that only single crystals with diffraction efficiencies above 1% (*e.g.* monochromator crystals, multilayer monochromators, *etc.*) and negligible dispersion effects can become problematic. In the case of the experiment presented further below, we detect 10^−2^ to 10^−3^ photons per single bunch, which is far below the detector limit. However, with the upgrade of the Elettra storage ring, we expect a significant increase in beamline brilliance, which could make the ‘single X-ray per pixel and bunch’ more relevant. Notably, the next detector generation with gating compatibility [Eiger2 (Dectris, Switzerland)] has a fivefold increase in pixel density, which will decrease the number of photons per pixel by 80% and hence help to mitigate possible detection limitations.

### 2.3. High-repetition-rate laser and synchronization   

In order to utilize the available single-pulse flux as efficiently as possible, we installed an Nd:YAG (1030 nm wavelength of fundamental laser radiation, 8 nm spectral FWHM) high-repetition-rate laser system (Light-Conversion, Lithuania), delivering femtosecond pulses (selectable, 240 fs to 10 ps). The laser power was chosen sufficiently high to obtain fluences of >1 mJ cm^−2^, even at short wavelengths (second and third harmonic at 515 and 343 nm, respectively) and high repetition rates (maximum 578 kHz, corresponding to half of the ring clock). Temporal synchronization of the laser with the storage ring (see Fig. 2 ▸) is achieved by phase locking the RF/6 = 83.3 MHz (12 ns) oscillator to the ring RF signal, using the commercially available phase-comparison system (PhaseLock, TEM-Messtechnik, Germany). The system also contains an electronic phase-counter, which allows automated control over laser delay in a range of 98 µs and in steps of 1.7 ps. For larger delays, *i.e.* experiments are performed with repetition rates <10.2 kHz, an external trigger is used to adjust the oscillator phase in full steps (12 ns), whereas fine-adjustment is made via the ‘PhaseLock’ module. Phase-noise measurements (MenloSystems, Germany) of the final setup showed an overall timing jitter between laser oscillator and storage ring RF of FWHM <0.4 ps and a maximum difference of 0.9 ps (fixed delay, measured over a time period of 30 min).

**Figure 3:**
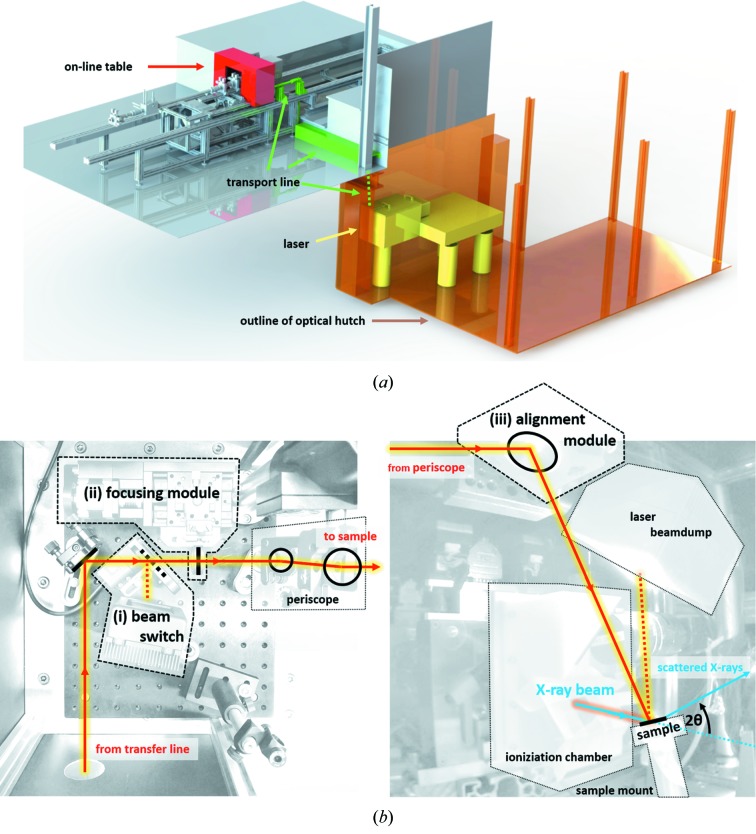
(*a*) Sketch of the laser infrastructure implemented at the Austrian SAXS beamline for pump–probe experiments. (*b*) Photographs of optical elements inside the on-line table enclosure in grazing-incidence configuration (left: top view; right: side view). Here, the laser trajectory is highlighted in red while the X-ray beam is shown in blue.

The laser was installed in a dedicated, newly constructed optical hutch [see Fig. 3(*a*) ▸] under class IV laser-safety regulations, including: a key-locked interlock system for access/operation control, fully light-proof enclosures, fast shutters for rapid intervention and redundant logic on all safety-related circuits. The laser beam is transported from the optical hutch to the X-ray sample stage via an optically sealed ∼5 m transfer line using six dielectric mirrors [see Fig. 3(*a*) ▸]. At the sample position, an enclosed and safety-locked optical table with pre-aligned optics may be placed on the beamline frame [see Fig. 3(*a*) ▸]. Three principle optical components are installed on this ‘on-line table’ and are motorized for remote-controlled operation [see Fig. 3(*b*) ▸]: (i) a laser shutter (controlling exposure of sample to laser beam with approximately 1 s response time), (ii) a linear-staged lens with focal distance depending on experimental needs (for fine-adjustment of the laser spot size on the sample), and (iii) a double-axis alignment mirror (required for spatial overlap of X-ray and laser beam). For grazing-incidence experiments, the laser beam is transferred above the sample position using a periscope system [see Fig. 3(*b*) ▸], such that the laser incidence angle can be adjusted in the range 20°–80° (but always perpendicular to the X-ray trajectory) by adequate positioning of the motorized mirrors. For transmission experiments, this motorized mirror can be installed instead of the upper segment of the periscope such that laser and X-ray beam lie within the same horizontal plane. In any case, a graphite beam-dump captures the reflected/transmitted laser beam above/behind the sample. A summary of the technical capabilities currently available at the beamline is given in Table 1 ▸.

## 3. Results   

### 3.1. Sample geometry and experimental conditions   

**Figure 4:**
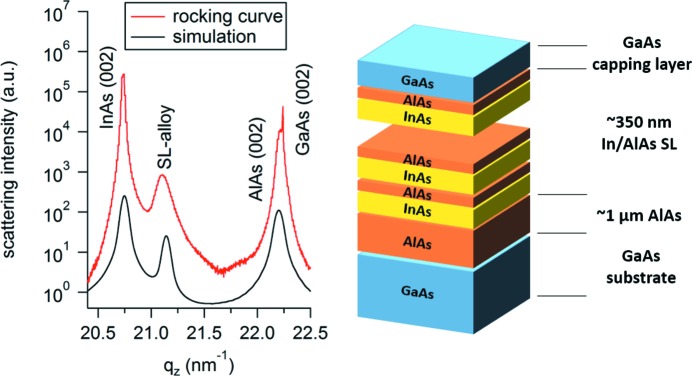
Rocking-curve (θ–2θ) scan of the In/AlAs superlattice sample (red) compared with the theoretical simulation of the design geometry (black). Here, the ‘SL-alloy’ peak is likely caused by scattering from mixed In_*x*_Al_1–*x*_As layers (with *x* ≃ 0.8) at the InAs/AlAs interface. A schematic overview of the sample geometry is shown on the right. *q*_*z*_ is the out-of-plane component of the scattering vector.

For the first proof-of-principle experiment, we designed a robust sample with known scattering behaviour for spatial and temporal overlap of X-ray and laser pulses: an In/Ga/AlAs superlattice (see Fig. 4 ▸). The design concept is based on the principle that a femtosecond laser pulse at 1030 nm is absorbed exclusively by InAs layers within the superlattice, leading to an instantaneous (sub-picosecond) heating of the crystal lattice [similar to Shayduk *et al.* (2011 ▸) and Schick *et al.* (2016 ▸)]. These local hot spots equilibrate by transferring the absorbed heat to the sandwiched AlAs layers, causing a fast InAs lattice relaxation within several hundreds of picoseconds after photo-excitation. The surface layers then slowly transfer the heat to the GaAs substrate – a process that is expected to occur on a >100 ns timescale. A corresponding 60 × InAs (3.63 nm)/AlAs (2.26 nm) superlattice was grown by molecular beam epitaxy on a (001) GaAs substrate (see illustrative representation in Fig. 4 ▸). To ensure better growth conditions, we added an approximately 1.2 µm AlAs layer as virtual substrate. A θ–2θ rocking-curve scan shown in Fig. 4 ▸ shows overall agreement of the real superlattice structure with the design geometry [theoretical calculations neglecting the GaAs substrate were performed using dynamical scattering theory, implemented in the udkm1Dsim toolkit (Schick *et al.*, 2014 ▸)].

**Figure 5:**
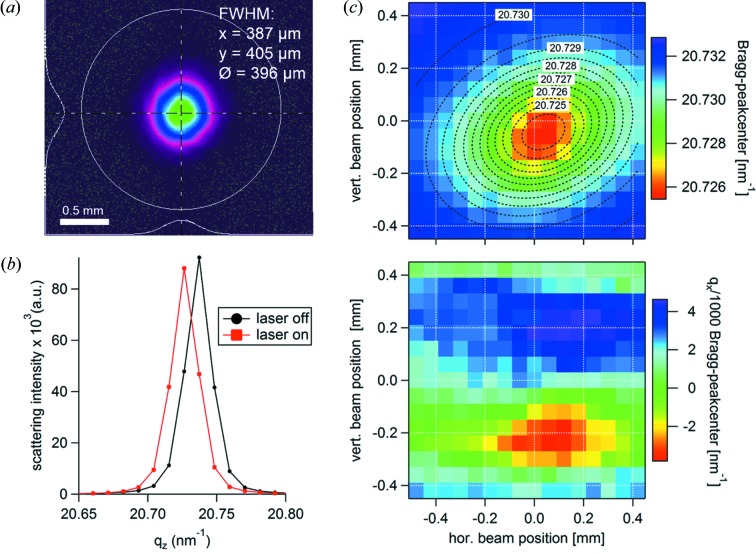
(*a*) Laser beam profile measured at normal incidence at the sample position (Spiricon, Ophir, Israel). The white lines show the horizontal [*x*] and vertical [*y*] Gaussian fits of the intensity profile. (*b*) Scattering patterns of the InAs peak with laser off (red) and on (black) at 14.723° X-ray incidence angle – the peak shift corresponds to a temperature increase of approximately 120 K. The experimental error given by Poisson counting statistics is <10^−3^ – error bars are hence omitted for clarity. (*c*) InAs Bragg peak centre (*q*_*z*_ component shown on top, the in-plane component *q*_*x*_ of the scattering vector shown on bottom) at different laser beam positions. The black dotted lines show the contours of the corresponding D-Gaussian fit.

The X-ray beam at 8 keV was reduced to a cross section of 0.2 mm × 0.1 mm (H × V), leading to an effective spot size on the substrate of 0.2 mm × 0.4 mm at 14.723° incidence angle [corresponding to the InAs (002) *d*-spacing]. The laser was set to an average power of 2.5 W at 1030 nm, with a repetition rate of approximately 385 kHz (corresponding to one-third of the ring clock to ensure thermal relaxation between pump pulses), thereby delivering pulses of approximately 6.4 µJ (measured at the sample position; Nova II, Ophir, Israel). The laser beam was purposely defocused (using a lens with 500 mm focal length) to result in a spot size at the sample of approximately 0.4 mm (FWHM, measured at normal incidence). As seen in Fig. 3(*b*) ▸, the laser beam impedes the sample at non-normal (approximately 20° from out-of-plane axis) incidence, such that the effective (slightly elliptical) spot size is approximately 0.43 mm, hence defining the fluence at 3.0 mJ cm^−2^ [see the Gaussian beam profile measurement after the transfer line in Fig. 5(*a*) ▸]. Under these conditions, no laser-induced sample damage was observed over >5 h of continuous exposure (checked by visual inspection under microscope). In order to assure thermalized and stable conditions under laser exposure, we waited >5 min after turning the laser light on before performing any alignment or pump–probe measurement (despite the fact that we observe no change in scattering behaviour after the first minute). In the following, we determine the position of the diffraction peak using the centre of mass method, as described in Appendix *A* [App appa].

### 3.2. Spatial overlap of X-ray and laser beam   

We determine the spatial overlap between X-ray and laser beam in a six-step process, similar to the protocol in the literature (Reinhardt *et al.*, 2016 ▸). Step 1: we pre-align the sample with its centre in the X-ray beam and bring it to the correct X-ray incidence angle with laser-beam off [here 14.723° corresponding to unstrained InAs (002) reflection]. A scattering image is taken as reference. Step 2: we turn on the laser with one-tenth of the final average power (= 0.25 W) to reduce the safety risk of damage through unwanted reflections. Step 3: we align the laser beam by a 2D ‘mirror alignment’ scan [see module (iii) in Fig. 3(*b*) ▸] and monitoring the Bragg peak positon.^1^ Step 4: we increase the laser power to the final target value (= 2.5 W). Step 5: we now align the laser beam by a 2D ‘mirror alignment’ scan and determine the exact position by a 2D-Gaussian fit [see Fig. 5(*b*) ▸]. The fitting result further reveals a beam overlap-area of 0.41 mm × 0.51 mm (H × V) due to the convolution of both beams. A scattering pattern taken at the centre position yields a peak shift of 0.011 nm^−1^ [see Fig. 5(*c*) ▸] compared with the reference (with laser off), from which a mean temperature rise in the sample of approximately 120 K can be estimated (Shayduk *et al.*, 2011 ▸; Levinshtein *et al.*, 1996 ▸). Step 6: we compensate for this expansion by readjusting the incidence angle to the maximum intensity of the now thermally strained InAs Bragg reflection (from 14.723° to 14.714°). The full alignment procedure consumes approximately 20 min – an overhead that is acceptable in order to ensure repeatability and stability of the experimental conditions.

### 3.3. Temporal overlap   

We determine the temporal overlap between laser and X-ray pulses by rough and fine scans of the laser delay. The measurement of a single diffraction pattern consists of 10^6^ gate-pulses and thereby single bunches, such that each image acquisition takes approximately 4 s. A rough delay scan over one entire pump–probe phase (3 × 864 = 2592 ns) in 10 ns steps hence consumes approximately 17 min. The full temporal overlap procedure includes three consecutive scans with increasing time resolution (10 ns, 200 ps and 5 ps), thereby taking approximately 1 h.

**Figure 6:**
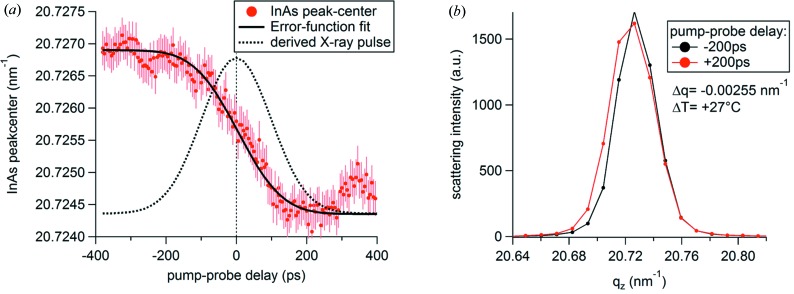
(*a*) InAs Bragg-peak position as a function of pump–probe delay (red). An error-function model (black) was fitted to the experimental data to determine the temporal overlap as well as the X-ray pulse width (see black dotted line for corresponding Gaussian approximation). The difference of fit and data between 300 and 400 ps stems from heat transfer in the sample. (*b*) Scattering patterns of the InAs (002) peak 200 ps before (red) and after (black) the pump pulse. The experimental error (contribution of Poisson counting statistics and standard deviation of frames within the X-ray pulse-width) is <1.7% – error bars are hence omitted for clarity.

The InAs peak centre over the variable pump–probe delay is shown in Fig. 6(*a*) ▸. From an error-function fit (Durbin *et al.*, 2012 ▸), we determine the zero-time delay as well as the temporal width of the X-ray pulse (235 ps FWHM). Indeed, single-scattering patterns taken 200 ps before and after the excitation [see Fig. 6(*b*) ▸] reveal a pump-induced peak shift of 0.0026 nm^−1^, which corresponds to a transient temperature jump of approximately 27 K (assuming instantaneous heating by pump).

### 3.4. Transient heat-transfer   

In order to capture the transient stages of heat transfer in the sample, we obtained further measurements at the InAs as well as at the GaAs Bragg condition (incidence angles of 14.714° and 15.785°, respectively). From the shift of the diffraction-peak positions, we calculate the transient temperature in the corresponding layers within the sample as a function of pump–probe delay [assuming linear, non-temperature dependent thermal expansion coefficients of α = 4.5 × 10^−6^ and 5.2 × 10^−6^ for InAs and GaAs, respectively (Levinshtein *et al.*, 1996 ▸)].

**Figure 7:**
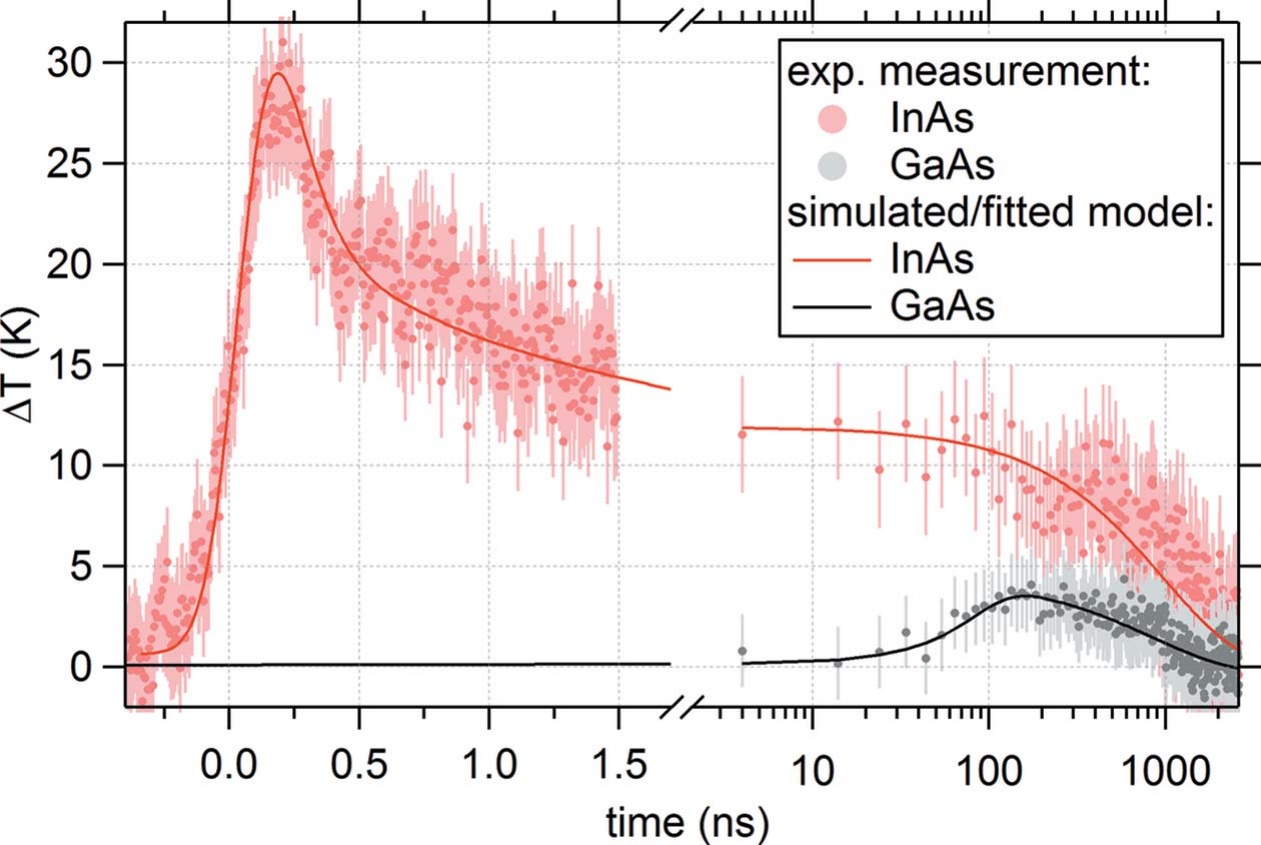
Transient heat-transfer through the InAs (red) and GaAs (black) layers, induced by 3.0 mJ cm^−2^ pulses at 1030 nm with 385 kHz repetition rate.

As seen in Fig. 7 ▸, the IR pulse causes an immediate heating of InAs within the superlattice, which rapidly transfers its heat to sandwiched AlAs layers (heating and partial cooling in less than 500 ps). Once the deposited heat has equilibrated in the surface-near superlattice at Δ*T* ≃ 12 K, a slow thermal diffusion process commences, causing an increase of the GaAs substrate temperature of approximately 5 K. A heat-transfer simulation based on the design geometry [see black curve in Fig. 4 ▸, simulated using udkm1Dsim^2^ (Schick *et al.*, 2014 ▸)] of the first 1.5 ns after excitation is in outstanding agreement with the experimental data (see red, solid line in Fig. 7 ▸). The longer phenomena (5–2500 ns) have been fitted using the single exponential cooling law (O’Sullivan, 1990 ▸) (a multiplied error-function term describes heat deposition in the substrate) – also here the excellent agreement with the experimental data shows validity of the chosen model and hence confirms transient heat diffusion away from the superlattice into the bulk substrate. The thermal constants and temperature-dependent equations for InAs, AlAs and GaAs used in the model simulations and calculations can be found in Table 2 ▸. Overall, the experimental results show successful use of the constructed laser-pump/X-ray-probe setup in a feasible acquisition time (approximately 4 h of exposure for all measurement shown in Fig. 7 ▸).

## 4. Summary and outlook   

We have successfully implemented a setup for picosecond pump–probe X-ray scattering at the Austrian SAXS beamline at Elettra-Sincrotrone Trieste. The modular design of the newly constructed beamline components allows a fast transition from ‘standard’ small- and wide-angle X-ray scattering measurements to laser-driven, time-resolved experiments within only a few hours. We further show that detection of single X-ray pulses in the Elettra hybrid filling mode is possible using a large-area, multi-panel detector and without installation of additional X-ray optics (*e.g.* chopper). A first test and reference experiment studying transient heat transfer in an In/Al/GaAs superlattice was successful. The pump–probe setup is available for user operation and will open a new chapter in the history of time-resolved experiments at the Austrian SAXS beamline.

Looking at developments in the next decade, the Elettra storage ring will be upgraded to a fourth-generation source. In the context of the new ring lattice, we expect to replace the current multi-pole wiggler insertion device with a new undulator, which will significantly increase the X-ray brilliance at the sample. Regarding improvements of the laser source, a beam-stabilization system is foreseen in the near future that will improve measurement conditions for acquisition-series taking longer than 4 h. Further, the design of the current setup, specifically the high laser-power as well as free space in the optical enclosure, already considers the addition of an optical parametric amplifier, which will allow more flexibility in tuning of the excitation wavelength.

## Figures and Tables

**Table:** 

Optical pump			
Wavelength (nm)	1030	515	343
Repetition rate (kHz)	1–600	1–600	1–600
Maximum average power (W)	20	10	6
Laser spot size (FWHM) (mm)	0.2–1	0.2–1	0.2–1
Pump fluence (mJ cm^−2^)	0.4–400	0.2–200	0.1–100
Polarization	*s*, *p*, elliptical, *c*+, *c*−		

**Table:** 

X-ray probe			
Incidence angles (°)	10	20	30
Horizontal spot size (mm)	0.02–0.8	0.02–0.8	0.02–0.8
Vertical spot size (mm)	0.02–0.15	0.02–0.3	0.02–0.45
Effective vertical spot size (mm)	0.1–0.85	0.06–0.85	0.04–0.85

**Table:** 

Energies (keV)	5.4	8	16
Incidence angles (°)	0–35	0–35	0–35
Possible *q*-range (nm^−1^)	0.01–25	0.03–38	0.07–60
Pixel resolution (nm^−1^)	0.002–0.03	0.004–0.05	0.007–0.09

**Table 2:** Material constants and equations used as input for the thermal response simulations and calculations Thermal constants were taken from Sealy (1993 ▸), Tiwari (1992 ▸) and Ng (2015 ▸), and optical constants from Adachi (1989 ▸) and Rakić & Majewski (1996 ▸).

	GaAs	InAs	AlAs
Absorption coefficient (cm^−1^)	1.8 × 10^3^	14.8 × 10^3^	3.4
Thermal conductivity (W K^−1^ m^−1^)	κ(*T*) = κ_300_(*T*/300[K])^−α^		
Constant κ_300_ (W K^−1^ m^−1^)	45.5	37	80
Exponent α	1.3	1.1	1.4
Specific heat (J K^−1^ kg^−1^)	*c*(*T*) = *c*_300_ + *c*_s_{[(*T*/300)^β^ − 1]/[(*T*/300)^β^ + (*c*_s_/*c*_300_)]}		
Constant *c*_300_	322	394	441
Constant *c*_s_	50	50	50
Exponent β	1.6	1.95	1.2

## Footnotes

1 Peak positions are determined by area-weighted numerical integration, as given in ISO Standard 11146.

2 Simulations were performed using dynamical scattering theory. Material properties such as density, specific heat capacity, temperature conductivity and thermal expansion coefficient were taken from Levinshtein *et al.* (1996 ▸). Refractive indices were calculated from dielectric functions from Aspnes & Studna (1983 ▸). Optical excitation of InAs was assumed to occur instantaneous (<1 ps). Note that the change in scattering signal is dominated by lattice expansion due to thermal heat transfer (the influence of acoustic phonon propagation is negligible in this time domain). For data comparison, the simulated diffraction patterns were evaluated using the identical procedure as the experimental data, leading to outstanding agreement of experiment and calculation.

## Appendix A Center of mass calculation

As seen in Figs. 5 ▸ and 6 ▸, the recorded diffraction peaks correspond to <10 detector pixels and hence *q*-bins in the scattering trace. To obtain a quantitative measure from such sparse data, we do not fit an analytical (*e.g.* Guassian) model, but determine the peak position by ‘centre of mass’ (CM) evaluation, according to

where *q*_*i*_ denotes the **q**-vector component (either *z* or *x*) corresponding to the experimental scattering intensity *I*_*i*_ and *N* denotes the number of points taken into account. To determine the error of the CM calculation, we apply standard laws of error-propagation yielding the standard deviation (SD) according to

where  denotes the experimental error of intensity *I*_*j*_ (Poisson statistics). This is valid under the assumption that *q*-binning is constant such that no stochastic (but possibly systematic) errors occur along the *q*-scale.
